# Anticancer effects of mifepristone on human uveal melanoma cells

**DOI:** 10.1186/s12935-021-02306-y

**Published:** 2021-11-17

**Authors:** Prisca Bustamante Alvarez, Alexander Laskaris, Alicia A. Goyeneche, Yunxi Chen, Carlos M. Telleria, Julia V. Burnier

**Affiliations:** 1 Experimental Pathology Unit, Department of Pathology, McGill University, Montreal, QC Canada; 2 Cancer Research Program, Research Institute of the McGill University Health Centre, Montreal, QC Canada; 3 Gerald Bronfman Department of Oncology, McGill University, Montreal, QC Canada

**Keywords:** Uveal melanoma, Mifepristone, Drug repurposing, Cancer therapy

## Abstract

**Background:**

Uveal melanoma (UM), the most prevalent intraocular tumor in adults, is a highly metastatic and drug resistant lesion. Recent studies have demonstrated cytotoxic and anti-metastatic effects of the antiprogestin and antiglucocorticoid mifepristone (MF) in vitro and in clinical trials involving meningioma, colon, breast, and ovarian cancers. Drug repurposing is a cost-effective approach to bring approved drugs with good safety profiles to the clinic. This current study assessed the cytotoxic effects of MF in human UM cell lines of different genetic backgrounds.

**Methods:**

The effects of incremental concentrations of MF (0, 5, 10, 20, or 40 μM) on a panel of human UM primary (MEL270, 92.1, MP41, and MP46) and metastatic (OMM2.5) cells were evaluated. Cells were incubated with MF for up to 72 h before subsequent assays were conducted. Cellular functionality and viability were assessed by Cell Counting Kit-8, trypan blue exclusion assay, and quantitative label-free IncuCyte live-cell analysis. Cell death was analyzed by binding of Annexin V-FITC and/or PI, caspase-3/7 activity, and DNA fragmentation. Additionally, the release of cell-free DNA was assessed by droplet digital PCR, while the expression of progesterone and glucocorticoid receptors was determined by quantitative real-time reverse transcriptase PCR.

**Results:**

MF treatment reduced cellular proliferation and viability of all UM cell lines studied in a concentration-dependent manner. A reduction in cell growth was observed at lower concentrations of MF, with evidence of cell death at higher concentrations. A significant increase in Annexin V-FITC and PI double positive cells, caspase-3/7 activity, DNA fragmentation, and cell-free DNA release suggests potent cytotoxicity of MF. None of the tested human UM cells expressed the classical progesterone receptor in the absence or presence of MF treatment, suggesting a mechanism independent of the modulation of the cognate nuclear progesterone receptor. In turn, all cells expressed non-classical progesterone receptors and the glucocorticoid receptor.

**Conclusion:**

This study demonstrates that MF impedes the proliferation of UM cells in a concentration-dependent manner. We report that MF treatment at lower concentrations results in cell growth arrest, while increasing the concentration leads to lethality. MF, which has a good safety profile, could be a reliable adjuvant of a repurposing therapy against UM.

**Supplementary Information:**

The online version contains supplementary material available at 10.1186/s12935-021-02306-y.

## Background

Melanomas are predominantly of cutaneous or ocular origin [1, 2] and present a host of distinct clinical challenges in relation to detection, treatment, and metastasis [3–5]. Ocular melanomas remain a diagnostic burden to oncologists as upwards of 83% arise in the vascular portion of the inner eye or uvea [1]. Uveal melanomas (UM) predominate in an inaccessible region, the choroid, growing undetected and often becoming highly metastatic [6, 7]. Despite effective local treatment, including plaque brachytherapy or enucleation [5, 8], up to 50% of patients develop metastases during the course of their lifetime [7, 9, 10]. Metastatic lesions emerge in the liver (89%), lung (29%), and bone (17%), and overall survival decreases below 20% within the first 2 years [9, 11–13].

To date, metastatic UM patients enter an abyss where a shallow understanding of their disease compounds the minimal efficacy of systemic treatment regimens. Clinically approved therapies in metastatic cutaneous melanoma, if applied to UM, have suboptimal or inconclusive results [3, 14]. For instance, checkpoint inhibitor (PD-1/ PD-L1 or CTLA-4) immunotherapies are emerging as a promising treatment in cutaneous melanoma; ipilimumab, an effective CTLA-4 inhibitor, has been FDA approved as treatment in metastatic cutaneous melanoma, yet it has dismal success rates of 0–5% in UM (reviewed in [14]). This can be attributed in part to the divergent biology, mutational profile and localization of cutaneous melanoma and UM metastases [11, 15, 16]. While metastatic disease in cutaneous melanoma follows through lymphatics, in UM disease, given the lack of lymphatics in the eye, metastasis occurs hematogenously, mainly in the liver (reviewed in [15]).

Unfortunately, liver metastases continue to be a challenge, resisting systemic therapies and reoccurring at unfavorable rates [11, 15]. Systemic combination chemotherapy regimens remain the gold standard for treatment of liver metastases; however, response rates are poor and dependent on individual patient variability [11, 15]. A 30-year study of 661 metastatic UM patients reported a 3-year survival rate of only 2.9% in patients with liver localized lesions compared to 19.8% in patients with extrahepatic metastasis. Dominant treatments were chemotherapy (50%), or combination of treatment modalities (34%), improving median survival from 1.7 months in absence of intervention to 6.3 months [11]. While preventative adjuvant therapies have shown little promise due to a combined lack of research, there is an unclear understanding of metastatic risk, and insufficient evidence that any one therapy can improve patient survival [15, 17, 18]. In short, the UM community of clinicians and researchers lack effective methods to mitigate the propagation of metastatic UM.

Mifepristone (MF) has drawn attention as a potential cancer treatment as its potent cytotoxic effects have been demonstrated to disrupt the growth of several cancer cell types [19–21]. MF was originally synthesized in the 1980’s as an antiglucocorticoid agent, yet due to its unexpected potent antiprogesterone activity, it was rapidly repurposed to the field of reproductive medicine for early termination of pregnancy, emergency contraception, and menstrual cycle regulation [22–25]. MF was further recognized for its ability to inhibit cell growth in endometriosis, uterine fibroids, and benign cases of meningioma; in cancer, MF demonstrated antiproliferative effects toward cells of cervical, breast, endometrial, ovarian, gastric, lung, brain, and prostate origin (reviewed in [26, 27]). These initial conclusions on MF’s anti-cancer effects were in the context of hormone sensitive tumors, however our group has proven its effectiveness regardless of progesterone, androgen, and estrogen receptor expression [28]. Moreover, we have shown that MF-induced growth inhibition is associated with blockage of the cell cycle and inhibition of DNA synthesis [20, 21]. The influence on cell proliferation is independent of the level of chemosensitivity or genetic background of the cancer cells [29, 30]. As a growth inhibitor, we have also shown that MF prevents the repopulation of cells that escape the lethality of chemotherapeutic agents such as cisplatin or paclitaxel [30–32].

In the present work, we evaluated the effect of MF on UM cells to establish whether the repurposing of this safe drug can become an effective adjuvant therapy for the treatment of UM. The anti-growth effect of MF was evaluated against a panel of human UM cells of different phenotypic origins and genetic backgrounds. We demonstrate that MF impairs the functionality, growth capacity, and viability of UM cells in a concentration-related manner. Lethal concentrations were associated with induction of caspase-3/7-related apoptosis and release of cell-free DNA. Further, we prove that the potency of MF observed in UM is unrelated to the expression of cognate progesterone receptors.

## Materials and methods

### Cell lines, culture conditions, and treatments

Primary human uveal melanoma cell lines MP41 and MP46 were acquired from the American Type Culture Collection (ATCC, Manassas, VA, USA). Primary MEL270 and metastatic OMM2.5 cell lines were kindly gifted by Dr. Vanessa Morales (University of Tennessee). Primary UM cells 92.1 were kindly gifted from Dr. Martine Jager [33]. MCF-7 breast cancer cells utilized as a positive control for classical progesterone receptor expression were obtained from ATCC. All previous cell lines were cultured in Roswell Park Memorial Institute media (RPMI 1640, Corning, Corning, NY, USA). Media was supplemented with 2 mM l-Alanyl-l-Glutamine (Glutagro, Corning), 100 U/ml penicillin and 100 µg/ml streptomycin (Corning), 10 mM HEPES (Corning), 10 μg/ml insulin (Roche, Basel, Switzerland), 1 mM sodium pyruvate (Corning), and 10% fetal bovine serum (FBS, Corning). Cells were kept at 37 ºC and 5% CO_2_ in a humidified incubator. Cell lines were authenticated by Short Tandem Repeat (University of Arizona Genetic Core).

Wild type choroidal melanocytes (wtCM) were isolated from donor eyes following a previously established protocol [34]; mutant CM (mutCM) carrying a point mutation [GNAQ(Q209L)] were generated from wtCM by viral transduction using plasmids psd44-GqQL, pMD2.G, and psPAX2 (Addgene, Watertown, MA, USA); the mutation reduces GTPase activity resulting in a constitutively active phenotype. Both wtCM and mutCM were cultured in serum-free melanocyte growth medium M2 (PromoCell, Heidelberg, Germany). Human eyes were used in accordance with the McGill University Health Centre (MUHC) Research Ethics Board (2019-5314).

Mifepristone (MF; Corcept Therapeutics, Menlo Park, CA, USA) was dissolved in dimethyl sulfoxide (DMSO) to generate a 40 mM stock solution that was stored at − 20 ºC. Prior to each experiment, the drug was thawed and freshly prepared in media to reach a final concentration of 5, 10, 20, or 40 μM. The final concentration of DMSO (Corning) in the media was 0.1% and served as vehicle control in the absence of MF.

### Cellular confluence

Cellular morphology and magnitude of confluence were assessed in real time using the Incucyte^®^ S3 Live-Cell Analysis System (Sartorius, Ann Arbor, MI, USA). Cells were seeded in 12-well plates (Corning) at 8 × 10^4^ cells per well for 24 h. Thereafter, cells were treated with 5, 10, 20, or 40 μM MF and placed in the Incucyte^®^ System. The software was adjusted to take 9 images per well every 6 h over the 72-h period of treatment. The Incucyte^®^ System phase contrast software provided an average percent confluence for each well. Cell proliferation is quantified by counting the number of phase objects overtime. Occupied area (% of confluence) represents cells imaged over time.

### Cellular functionality

1.5 × 10^4^ cells per well were seeded in a 96-well plate (Corning) 24 h prior to treatment. Cells were kept under 5, 10, 20, or 40 μM MF treatment for 72 h. 10 μl of cell counting kit 8 solution (CCK8, Dojindo Molecular Technologies, Kumamoto, Japan) was added. After 1 h of incubation at 37 °C and 5% CO_2_, absorbance was read at 450 nm using an Infinite M200 Pro plate reader (Tecan Trading AG, Männedorf, Switzerland). Cells with no treatment were used as a negative control. Media and CCK8 solution in the absence of cells were used as a blank control. Percentage of metabolic activity was calculated according to the following equation: sample – blank/negative control − blank × 100.

### Trypan blue exclusion test

2 × 10^5^ cells per well were seeded in a 6-well plate (Corning) 24 h prior to treatment. Cells were then exposed for 72 h to 5, 10, 20, or 40 μM MF. Thereafter, the cells were dissociated by enzymatic solution (0.05% trypsin, Corning), and 10 μl of cell suspension were mixed with 10 μl of 0.4% trypan blue solution (Corning). The number of dead and live cells was measured using a TC20 automated cell counter (Bio-Rad Laboratories, Hercules, CA, USA).

### Recovery assay

Cells were seeded at a density of 9 × 10^4^ cells per well in a 12-well plate (Corning), and treated with varying concentrations of MF (0, 20, or 30 μM). Throughout the 72-h treatment period, cells were imaged every 6 h using the Incucyte^®^ S3 Live-Cell Analysis System at 4 × magnification. Following the initial 72 h of treatment, media was aspirated, and fresh media lacking MF was added to all wells. Cells were then imaged for a subsequent period of 72 h to assess their recovery capacity. Images obtained were then analyzed by the Incucyte^®^ S3 Live-Cell Analysis software, and cellular confluence data was exported for further quantitative analysis.

### Cell cycle analysis

After MF treatment, single cell suspensions were fixed with 4% paraformaldehyde (PFA) at room temperature for 1 h. Suspensions were centrifuged at 300 g for 5 min and pelleted cells were washed twice with phosphate-buffered saline (PBS). A suspension containing 2 × 10^5^ cells were re-pelleted and resuspended in 0.2 ml of propidium iodide (PI) solution containing 7 U/ml RNase A, 0.05 mg/ml PI, 0.1% v/v Triton X-100, and 3.8 mM sodium citrate (Sigma Chemical Co., St. Louis, MO, USA) for 20 min at room temperature or overnight at 4 °C protected from light. Cells were analyzed with the Guava Muse Cell Analyzer (Luminex Corporation, Austin, TX, USA), that takes advantage of the capacity of PI to stain DNA allowing detecting different DNA contents along the cell cycle. The cell cycle application of the Muse software was used to analyze the results and to determine relative stages of the cell cycle.

### Flow cytometric assessment of cell death

Early apoptosis and late apoptosis as well as necrosis were evaluated using the Dead Cell Apoptosis Kit with Annexin V-Fluorescein isothiocyanate (FITC) and PI, for flow cytometry double labelling (Thermo Fisher Scientific, Waltham, MA, USA) following the manufacturer’s instructions, and then analyzed in a BD FACSCanto II Cell Analyzer (BD, Evembodegem, Belgium). Cells staining with Annexin V-FITC without PI were considered early apoptotic, cells with double staining were considered late apoptotic, whereas cells that incorporated only PI were considered necrotic.

### DNA fragmentation

In a 100-mm dish, 1 × 10^6^ cells were seeded and cultured for 24 h, and then treated with MF for 72 h. Genomic DNA (gDNA) was isolated following a previous described protocol [35]. gDNA was separated in 2% agarose gels, stained with SYBR Gold nuclei acid stain (Thermo Fisher), and visualized in a ChemiDoc MP (Bio-Rad Laboratories).

### Caspase-3/7 activity

Cells were plated in a 96-well plate at 2 × 10^3^ cells per well and incubated for 24 h to allow attachment. MF treatment was added in a 1 × medium containing Essen Bioscience Incucyte^®^ Caspase-3/7 activity reagent (Sartorius, Ann Arbor, MI, USA). The caspase-3/7 dye crosses the cell membrane and is specifically recognized and cleaved by activated caspase-3/7 resulting in the release of a dye that binds to DNA and fluoresces green. The 96-well plate was placed in the Incucyte^®^ Live-Cell analysis system for live cell imaging for 72 h. Images from the scan interval were analyzed in the IC Incucyte^®^ software.

### Cell free DNA detection

Cell-free DNA (cfDNA) was detected using a known mutation in the UM cell lines. First, 3 × 10^5^ cells were seeded in a 6-well plate. Following 72 h of MF treatment at concentrations of 5, 10, 20, or 40 µM, 3 ml of culture supernatant was collected and spun at 300 g for 5 min. cfDNA was isolated using the QIAamp Circulating Nucleic Acid kit (QIAGEN, Hilden, Germany) following the urine protocol. cfDNA was kept in AVE buffer (RNase-free water with 0.04% sodium azide; QIAGEN), and quantified by a fluorometric method using a Qubit 4 (Thermo Fisher). Droplet digital PCR (ddPCR) (Bio-Rad Laboratories) was performed to measure the number of copies of cfDNA using wild type sequences and hotspot mutations GNAQ (Q209L and Q209P) and GNA11 (Q209L) by following a previously reported protocol [36]. No template control was added in each assay. Individual runs were performed in triplicates.

### Quantitative real-time reverse transcriptase (qPCR)

Gene expressions of progesterone receptor (PR), progestin and adipoQ receptor family member 8 (PAQR8), membrane-associated progesterone receptor component 1 (PGRMC1), and component 2 (PGRMC2), the glucocorticoid receptor: receptor subfamily 3 group C member 1 (NR3C1), and β-Actin (as a reference gene) were quantified using SybrGreen-based Real Time PCR in a CFX96 Touch Real Time PCR detection system (Bio-Rad Laboratories). qPCR reactions were conducted in 20 μl volume for 40 cycles at 61 °C annealing temperature using SsoAdvanced Universal SYBR Green supermix (Bio-Rad Laboratories) and primers (ID Technologies, Coralville, IA, USA). RNA was extracted using the RNeasy Plus Micro kit (QIAGEN). Complementary DNA (cDNA) was synthetized using iScript (Bio-Rad Laboratories). The MCF-7 cell line was used as a positive control for the expression of classical progesterone receptor mRNA. No template control and no reverse transcriptase control were added in each assay. Individual runs were performed in triplicates. Data was analyzed using CFX Maestro Software (Bio-Rad Laboratories).

### Statistical analysis

Experiments were conducted at least three times in biological and technical replicates for each cell line. Results are expressed as the mean ± SD. Graphpad Prism 9 (Graphpad Software, La Jolla, CA, USA) allowed for statistical analysis of data using repeated measures two-way ANOVA followed by Tuckey’s multiple comparison test, two-way ANOVA followed by Dunnett’s multiple comparison test, or Student’s *t*-test depending on the experiment. Differences were significant if p < 0.05.

## Results

### Mifepristone inhibits functionality, growth capacity, and viability of human primary and metastatic UM cell lines in a concentration-related manner

**Fig. 1:**
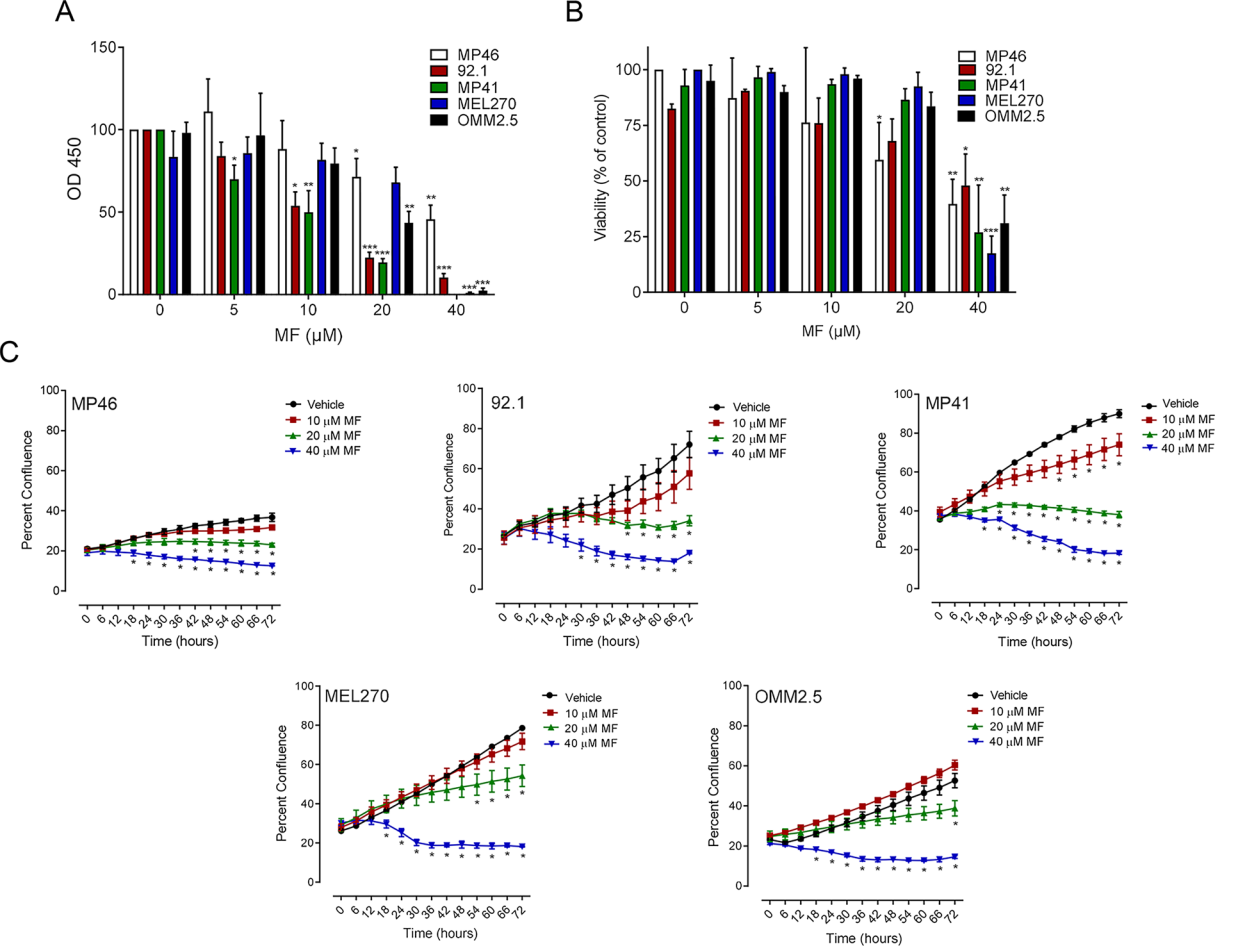
MF inhibits functionality, growth capacity, and viability of UM cell lines in a concentration-related manner. Graphs represent the level of cellular functionality or viability, respectively as detected via a CCK8 colorimetric assay (**A**) or Trypan Blue exclusion assay (**B**) after cells were treated with increasing concentrations of MF (0, 5, 10, 20, or 40 μM) for 72 h. **C** Growth curves obtained through Incucyte live cell imaging system, tracking cellular confluency. In **A** and **B**, data were analyzed using two-way ANOVA followed by Dunnett’s multiple comparison test. In **C**, data were analyzed using repeated measures ANOVA followed by Tuckey’s multiple comparison test.* Indicates p < 0.05, ** indicates p < 0.01, whereas *** indicates p < 0.001 compared against vehicle-treated controls

To determine whether MF treatment influences the functionality and viability of UM in vitro, a range of human primary UM cell lines (MP46, 92.1, MP41, MEL270) and a metastatic UM line (OMM2.5) were investigated. Cells were treated with increasing concentrations of MF (0, 5, 10, 20, or 40 μM), and incubated over a period of 72 h. A colorimetric assay, in which reduction of water-soluble tetrazolium salt (WST-8) produces orange formazan, was utilized as a means to determine metabolic activity of UM cells upon MF treatment. A concentration-dependent decrease in cellular dehydrogenase activity was observed for all UM cell lines (Fig. 1A). For concentrations of 5 to 40 μM of MF, UM cell lines all demonstrated reduction in functionality and significant cytotoxicity at 40 μM. To quantify the live cells in each sample and investigate late-stage cell death through disturbances in membrane permeability, a trypan blue exclusion assay was conducted. Concentrations of 5, 10 or 20 μM of MF resulted in no decrease in cellular viability except in MP46 cells that showed statistical significant reduction in viability upon incubation with 20 μM MF; 40 μM concentrations of MF caused a significant reduction in live cell count in all UM lines tested (Fig. 1B). To determine how MF affects population doubling of UM cell lines, cells were treated and imaged at 6-h intervals in the Incucyte live cell-imaging incubator over 72 h. We report a concentration-related reduction in cellular confluence across all UM cells (Fig. 1C). A significant deviation in cellular confluence was noted at 10 μM MF for MP41 cells only; 20 μM and 40 μM MF reduced confluence in MP41 as well as MEL270, 92.1, OMM2.5 and MP46 cells. A concentration of 40 μM had the strongest effect showing plateauing of the growth curves. As a visual example of the effect of MF we present in Additional file 1: Fig. S1 how increased concentrations of MF cause a decrease in the confluence of MP41 cells when using label free-phase masking quantification; the toxicity of MF is also revealed in the rounding and detachment of cells with the highest concentration of the drug. Wild type primary choroidal melanocytes (wtCM) isolated from donor eyes were used as control cells to estimate effects of MF on potential adjacent normal tissue. These wtCM display a steady confluence over time, which was not affected by concentrations of MF up to 20 μM (Additional file 2: Fig. S2A). When however the CM carry a point mutation [GNAQ(Q209L)] (mutCM), the cells acquired growth advantage reflected in their slight yet consistent increased confluence over time of incubation when compared to wtCM; in this case 20 μM MF did inhibit such sustained yet slow growth (Additional file 2: Fig. S2B).

### High concentrations of mifepristone cause permanent impairment in the proliferative abilities of UM cells

**Fig. 2:**
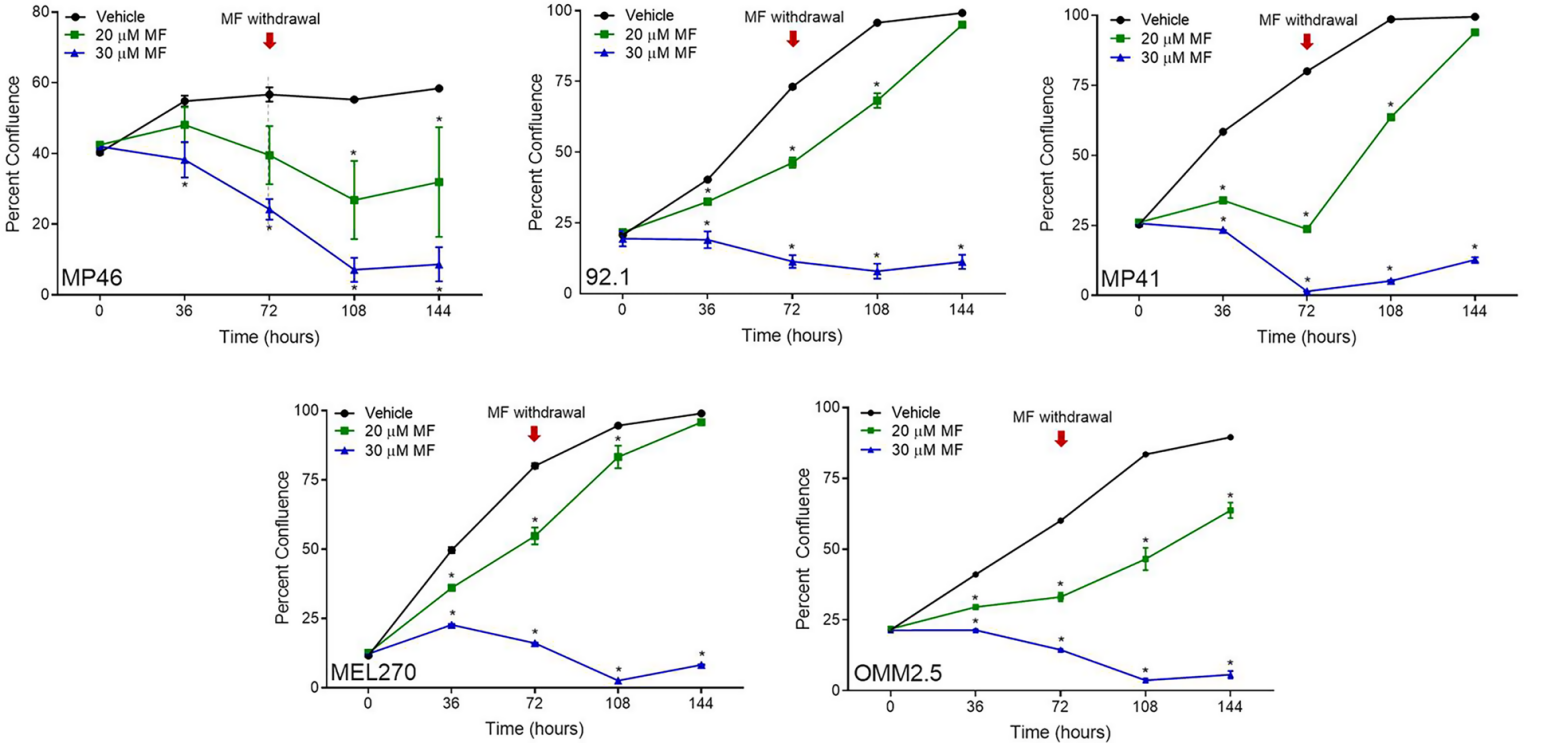
Long-term toxicity of MF towards UM cell lines and the consequence of MF withdrawal. UM cells were treated with MF at concentrations of either 0, 20, or 30 μM for 72 h and imaged every 6 h in the Incucyte. Following the initial 72 h, media was aspirated, replaced with regular growth media, and placed back into the Incucyte to be imaged for another 72 h. The red arrows at 72 h indicate the moment in which MF was removed from the media. Data were analyzed using repeated measures ANOVA followed by Tuckey’s multiple comparison test. * Indicates p < 0.05, ** indicates p < 0.01, whereas *** indicates p < 0.001 compared against vehicle-treated controls

To assess whether MF treatment has a long-term impact on cellular proliferation, we performed a recovery assay. Following treatment with 20 or 30 μM MF for 72 h, drug-supplemented media was replaced with regular growth media and cells were left to grow for another 72 h. All UM-cell growth curves significantly deviated from control, plateaued, or declined at concentrations of 20 or 30 μM during the initial period of 72 h period of incubation. Once MF treatment was withdraw at 72 h, cells either were able to partially, or totally repopulate the culture regaining confluence. In contrast, the confluence of cell populations treated with 30 μM MF did not recover regardless of the cell line studied (Fig. 2).

### Mifepristone at higher concentrations triggers accumulation of hypo-diploid DNA content, fragmented DNA, and of cells undergoing apoptosis

**Fig. 3:**
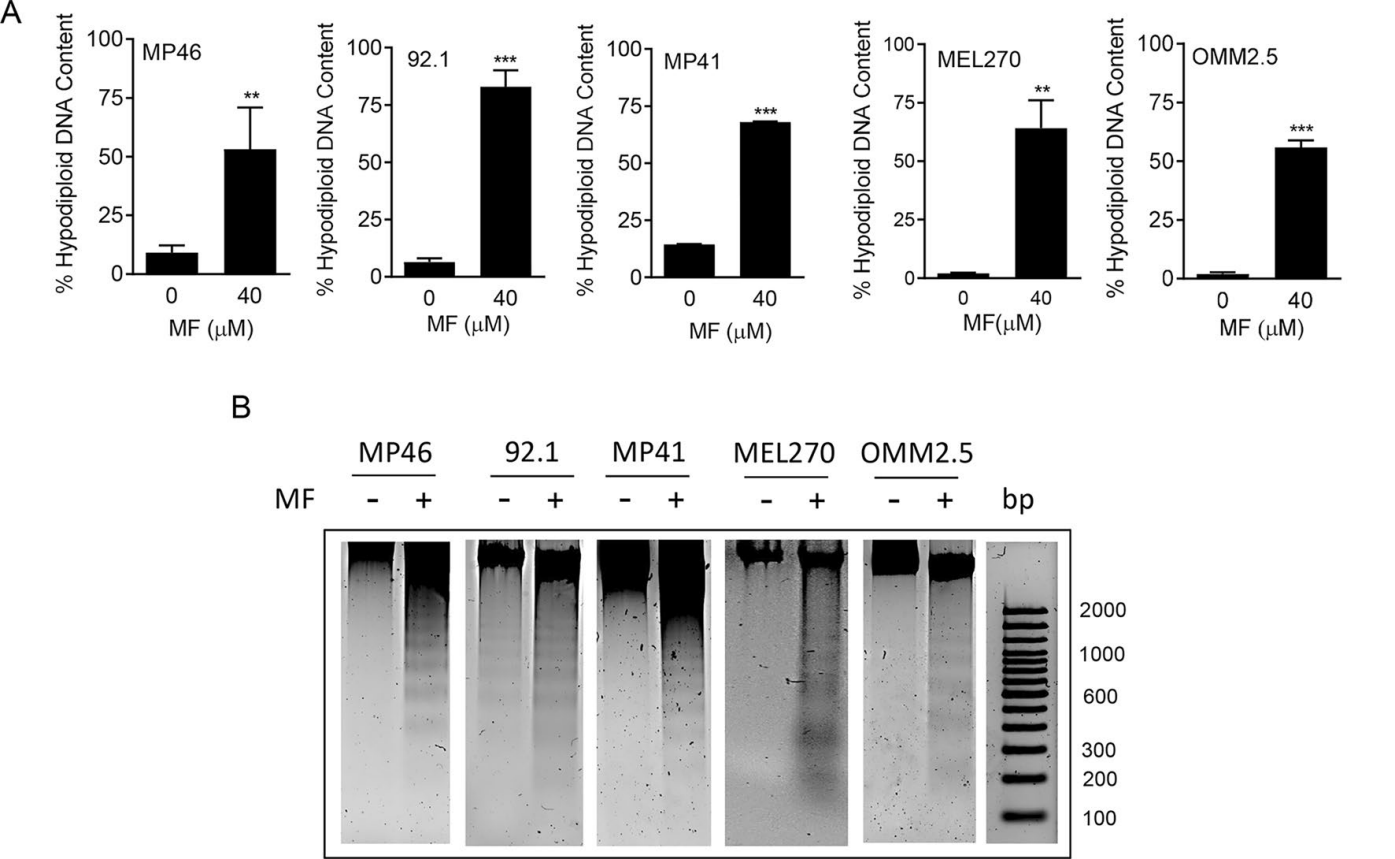
MF induces accumulation of hypodiploid DNA content and DNA ladder. **A** Quantification of particles with hypodiploid DNA content upon 72 h of MF treatment in a panel of UM cell lines. The hypodiploid DNA content corresponds to the Sub-G1 DNA content extrapolated when performing the cell cycle analysis of the cells treated with MF (the quantitative details are shown in the green-stained sections of the histograms in Additional file 3: Fig. S3). **B** A similar experiment was done in which all floating and adherent cells were pelleted, gDNA isolated, subjected to agarose electrophoresis, stained with SYBR Gold nuclei acid stain, and imaged. A 100 base pair (bp) maker was run in parallel. −: vehicle; + : 40 µM MF

To determine the extent to which MF causes cytotoxicity, we quantified the particles with hypodiploid DNA content, which coincides with DNA located in the Sub-G1 region of the cell cycle histograms. No rise in hypo-diploid DNA content was observed in cells treated with MF at concentrations ranging from 5 to 20 μM (data not shown). In contrast, a large increase in hypo-diploid DNA content was observed in all UM cell lines treated with 40 μM MF (Fig. 3A). When gDNA isolated from 40 µM MF-treated cells were run in agarose gels, we observed that the DNA shows fractionation typical of cells that are undergoing apoptotic cell death (Fig. 3B). In Additional file 3: Fig. S3 we clearly observe how the Sub-G1 region of the cell cycle histogram increases with the concentration of 40 µM MF, in all UM cell lines, when compared to the histograms displayed by cells receiving vehicle or 20 µM MF; of interest, the latter have a tendency of accumulating cells in the G1 phase of the cell cycle, yet without reaching statistical significance.

**Fig.4:**
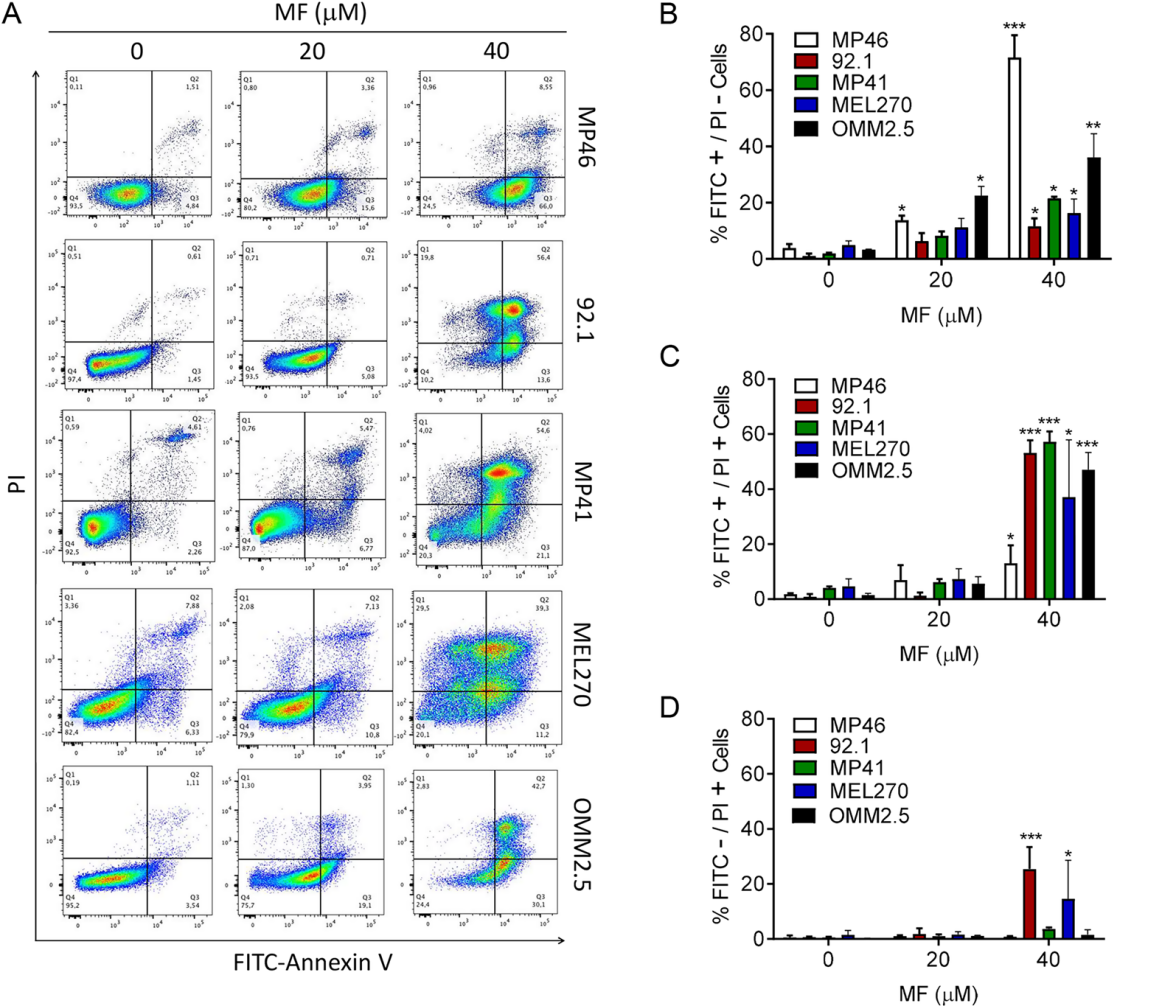
MF induces apoptosis in UM cells. **A** Representative histograms depicting the distribution of UM cells exposed to vehicle, 20, or 40 µM MF, and stained with Annexin V-FITC and/or PI after 72 h of incubation. The histograms represent flow cytometry data. **B** The bar graphs depict the percent of UM cells undergoing early apoptosis as marked by the labeling with only Annexin V-FITC. **C** Results show the percent of UM cells undergoing late apoptosis represented by cells double labeled with Annexin V-FITC and PI. **D** The percent of cells likely undergoing necrosis is shown as PI only stained cells. Data were analyzed using two-way ANOVA followed by Dunnett’s multiple comparison test. * Indicates p < 0.05, ** indicates p < 0.01, whereas *** indicates p < 0.001 compared against vehicle-treated controls

To further examine whether gDNA fragmentation and hypo-diploid DNA content accumulated in response to lethal concentrations of MF involves an apoptotic process, we incubated all UM cell lines with vehicle, 20 or 40 µM MF, and subjected them to double labeling with Annexin V-FITC conjugate and propidium iodide (PI). Figure 4A depicts the flow cytometric histograms of each one of the UM cell lines treated with vehicle or MF. Figure 4B shows the quantification of early apoptosis denoted by cells binding only Annexin V-FITC; Fig. 4C shows that only two cell lines, 92.1 and MEL270, display some level of necrosis as denoted by the cells binding PI.

### Lethal concentrations of mifepristone activate executer caspase-3/7

**Fig. 5:**
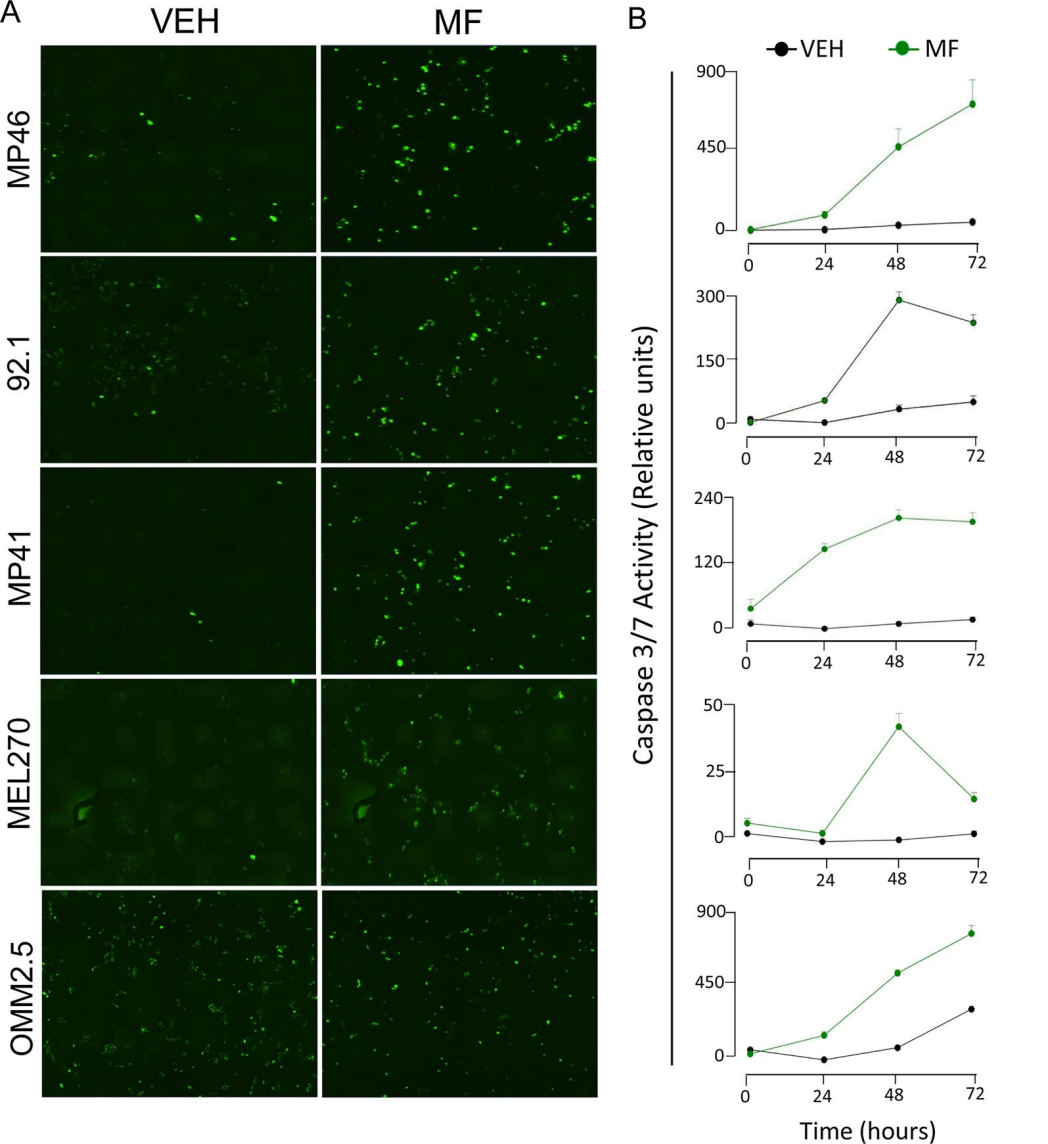
MF-associated UM cell death is related with the activation of executer caspase-3/7. **A** Green nuclear staining is generated upon a chemical reaction catalyzed by either active caspases 3 or 7. The images shown represent the endpoint of an experiment done for 72 h following MF treatment at a 40 µM concentration. These images can be observed over imposed with phase contrast in Additional file 5: Fig. S5. **B** Depicted are the time-course quantifications of the green fluorescence expressed as relative activity with respect to the fluorescence generated by vehicle-treated cells

To assess whether apoptosis induced by lethal concentrations of MF in UM cells involves activation of executer caspases, we studied the activation of caspases 3 and 7 following 72 h of treatment with either vehicle or 40 µM MF by using the Essen Bioscience Incucyte^®^ Caspase-3/7 activity reagent. Caspase-3/7 activities were found highly increased by the lethal concentrations of MF in all UM cell lines studied (Fig. 5A, B), as well as in wtCM and mutCM (Additional file 4: Fig. S4). The green fluorescence cellular content denoting caspase-3/7 activation in all cell lines shown in Fig. 5 can also be observed overlaid with phase contrast imaging (Additional file 5: Fig. S5).

### Mifepristone treatment induces the release of cell-free DNA into the culture media

**Fig. 6:**
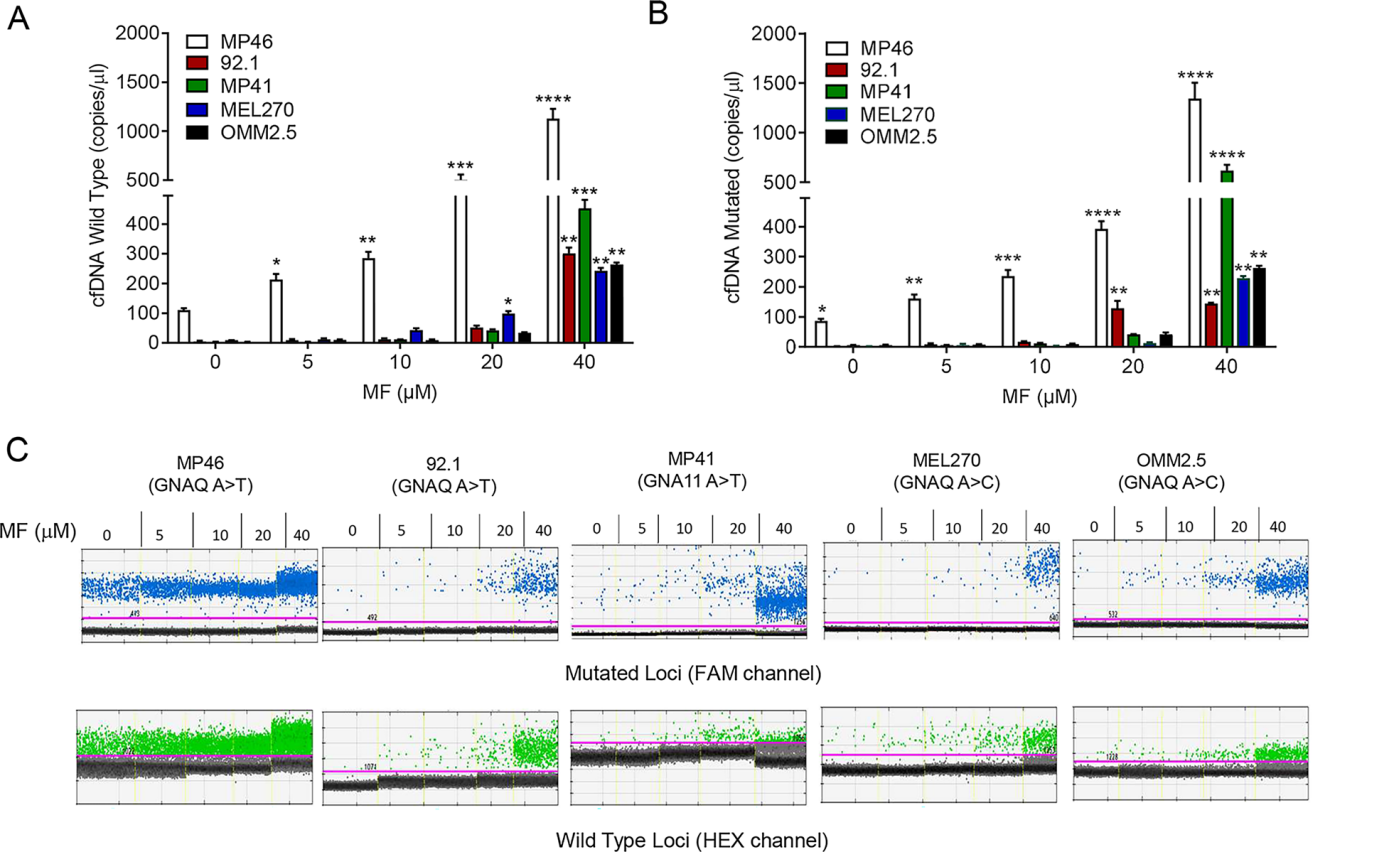
MF treatment induces the release of cell-free DNA into the media supernatant. Graphs show number of wild type (**A**) and mutant (**B**) copies of cfDNA per μl of cell-free media obtained 72 h after incubation with vehicle, 5, 10, 20, or 40 µM MF. **C** Representative one-dimensional plot of mutant GNAQ/GNA11 or wild type cfDNA extracted from conditioned media after treatment for 72 h with the depicted concentrations of MF. Channel compatible with FAM dye shows droplets with mutant target in blue. Wild type target is shown in green using a HEX label. Threshold (pink line) set in between positive (mutant or wild type) and no DNA target (black) droplets

Various studies have shown cell-free DNA (cfDNA) to be released from cells undergoing cell death [37]. We have previously shown the ability to utilize driver mutations in UM (GNAQ and GNA11 c626A > T and A > C) to detect and monitor circulating tumor DNA (ctDNA) in UM cell lines following drug treatment [36]. Here, we evaluated the release of GNAQ mutant (MP46, 92.1, MEL270, OMM2.5) and GNA11 mutant (MP41), as well as GNAQ/11 wild type cfDNA in the absence or presence of increasing concentrations of MF using ddPCR. After 72 h of MF treatment, we detected a concentration-dependent increase in both wild type and mutant cfDNA (Fig. 6A, B). The number of mutant and wild type copies detected upon treatment of each UM cell line with increasing concentrations of MF are depicted in Fig. 6C; they clearly denote a highly significant increase in cfDNA at the lethal concentration of 40 µM MF. Of interest, release of cfDNA was noted in MP46 and 92.1 cells in response not only to lethal concentrations of MF, but also to non-lethal ones.

### Mifepristone does not require the presence of classical nuclear progesterone receptors to inhibit growth and kill UM cells of different genetic backgrounds

**Fig. 7:**
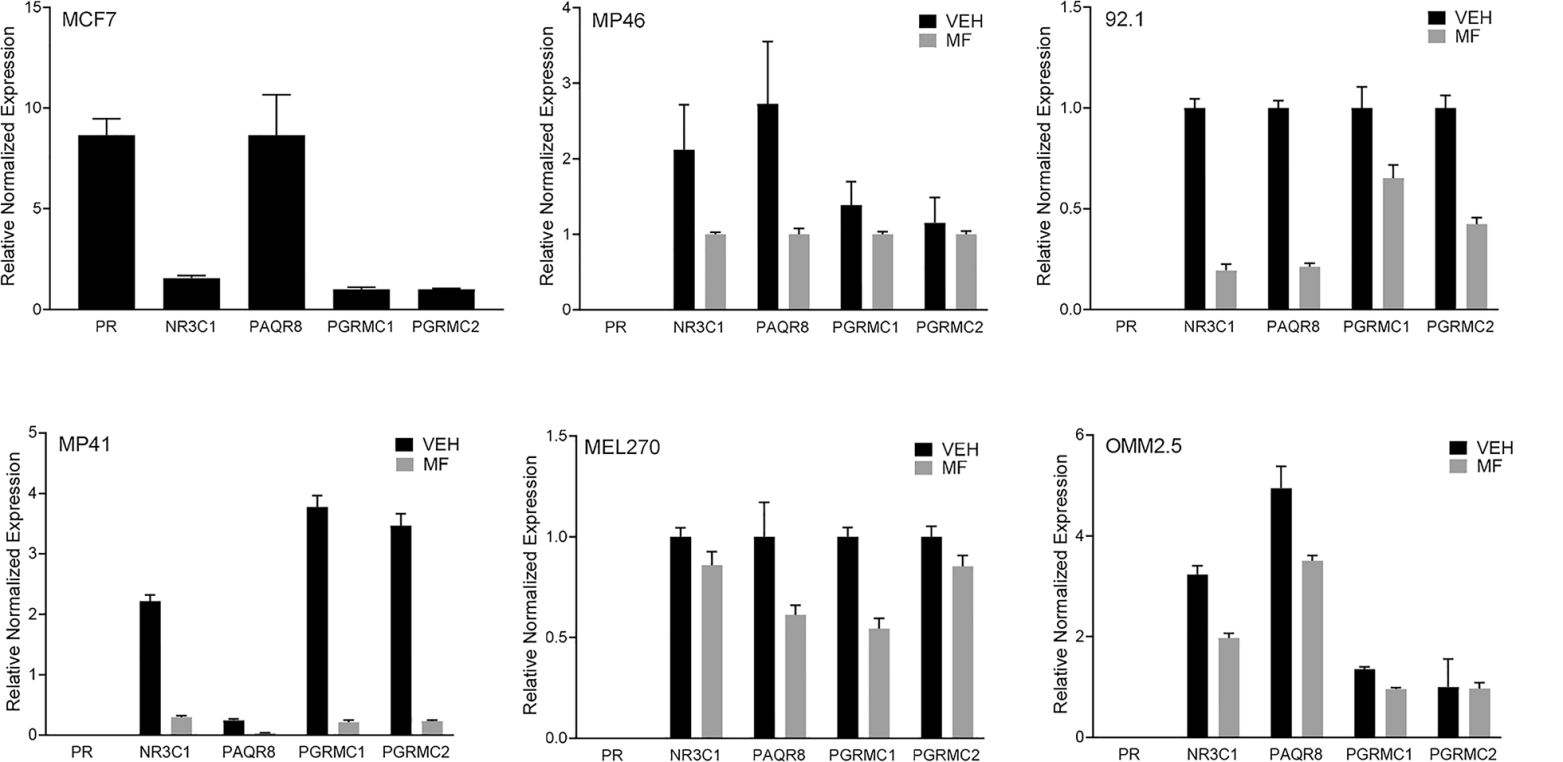
The effect of MF in UM cells is independent from the classical nuclear progesterone receptor**.** SybrGreen-based Real Time PCR quantified the gene expression profiles of PR, PAQR8, PGRMC1, PGRMC2, and NR3C1. β-Actin was used as a reference gene. mRNAs were amplified from either untreated cells or cells treated with 20 µM MF. The mRNA from MCF-7 cells was used as a positive control for the expression of the classical PR. No template control and no reverse transcriptase control were added in each assay. Individual runs were performed in triplicates

It has been previously demonstrated that the antiproliferative action of MF can be independent of the presence of nuclear progesterone receptor (PR), and instead be potentially mediated by membrane progesterone receptors or glucocorticoid receptors [28] (reviewed in [27]). To investigate whether UM cells express cognate progesterone receptors or the other alternative putative receptors, we analyzed their mRNA expression. This included the cognate progesterone receptor (PR), progestin and adipoQ receptor family member 8 (PAQR8), membrane-associated progesterone receptor component 1 (PGRMC1), and component 2 (PGRMC2), as well as the glucocorticoid receptor subfamily 3 group C member 1 (NR3C1). We used MCF-7 breast cancer cells as a positive control for the expression of the cognate PR [28]. qPCR results indicate that primary MP41, MP46, 92.1, and MEL270 cells, as well as metastatic OMM2.5 cells, all express the glucocorticoid receptor (NR3C1). Furthermore, all UM cells express non-classical progesterone receptors (PAQR8, PGRMC1, PGRMC2); however, the cognate nuclear PR is absent in all UM cells. Of interest, of the detected receptor’s mRNAs, all are downregulated in the presence of MF (Fig. 7).

## Discussion

There is a clear gap in treatment options that succeed in mitigating the progression of metastatic UM and ameliorate the survival of patients. Our group elected to improve this current situation by determining whether the promising literature on MF as an anti-cancer agent held in the context of UM. At concentrations of 10 μM and higher, MF significantly disturbed the natural proliferative curves of human UM cell lines. This potent inhibition in proliferation was accompanied by a significant reduction in cellular viability. We found the lower concentrations studied—5 μM—to affect the metabolic activity of the cells while higher concentrations resulted in disruption of membrane permeability, associated with later stage cell death. These results were consistent across all cell lines tested, including the highly metastatic line OMM2.5. The results found in UM cells are in line with previous reports in ovarian, cutaneous melanoma, and various additional cancer types [20, 28, 38].

MF has potent actions independently from cell line donors, clinical history, or mutational signatures. From the five UM cell lines in our study, 92.1 and MEL270 were originally derived from primary UM patient tumors [39]. The donor for line 92.1 presented with a large primary mass which resulted in complete exenteration of the orbit due to extension into rectus muscles [33, 39]. In contrast, MEL270 cells were obtained following enucleation of a patient who had previously undergone plaque irradiation treatment for primary UM. The cell line OMM2.5 was cultured from liver metastases discovered in the same patient, making MEL270 and OMM2.5 a primary and metastatic donor matched pair [39, 40]. Finally, MP41 and MP46 were cultured from patient derived xenografts of primary UM [41]. All cell lines tested were susceptible to the toxicity of MF in a concentration-related manner.

UM is characterized by mutually exclusive early guanine nucleotide-binding protein alpha Q (*GNAQ*) or alpha 11 (*GNA11*) activating mutations present in each of the cell lines studied here [42, 43]. Moreover, our panel of UM cell lines covers a variety of additional and differential mutational statuses. For example, MP46 has loss of heterozygosity of chromosome 3 and no BRCA-1 associated protein 1 (BAP1) expression, both associated with increased metastatic risk and function as prognostic indicators of metastasis [5, 44–46]. In contrast, cell line 92.1 has disomy 3 and a eukaryotic translation initiator factor 1A X-linked (*EIF1AX*) mutation [39, 41], both correlated with a significantly lowered risk of metastasis [44, 47]. Regardless of genetic background and metastatic potential, MF influenced the growth and viability of all cell lines in a relatively similar manner.

The MF-induced growth inhibition observed in UM is consistent with that observed in other cancer types [21]. We demonstrated that at lower concentrations, MF induced a cytostatic effect in UM, while higher concentrations resulted in high cytotoxicity associated with reduction in cellular viability. A MF-dependent decrease in cyclin dependent kinase-2 (Cdk2) was suggested as the mechanism underlying growth arrest. We previously demonstrated an increase in the abundance of cell cycle inhibitors p21^cip1^ and p27^kip1^ with a simultaneous decrease in Cdk2 activity and cyclin E abundance, all supporting the notion that MF blocks cell cycle progression at the G1/S transition [20, 21, 28].

In terms of the lethality caused by MF at higher concentrations in UM cells, we found that the most prominent effect was the double labeling of the cells with Annexin V-FITC and PI indicating that the majority of the cells, upon 72-h incubation with MF, are in a stage of late apoptosis. Nevertheless, we found that the slowest proliferating cells, MP46 and OMM2.5, showed signs of early apoptosis as marked by Annexin V-FITC-only labeling at the 20 μM concentration of MF. In addition, we found two cell lines (92.1 and MEL270) with a very slight proportion of cells undergoing necrosis associated with apoptosis. The concomitant accumulation of hypodiploid-DNA content, DNA fragmentation, and double labelling Annexin V FITC-PI, denotes that UM cells treated with lethal concentrations of MF mostly die by a classical process of apoptosis. This apoptosis also is associated with the activation of executer caspase-3/7. We have shown that MF causes lethality of other cancer cell types associated with accumulation of cells with hypodiploid DNA content and DNA fragmentation [21, 31]. Given that UM usually presents with a phenotype not very prone to undergo apoptosis [48], manipulation of proapoptotic pathways using an external agent such as MF may represent a potent therapeutic approach for the management of this disease.

During cellular death or cancer progression, the release of highly fragmented cfDNA is amplified, and can be detected in bodily fluids. cfDNA is mainly released through processes of apoptosis, necrosis and cellular secretions, and can inform us of the current state of a tumor or cellular system [49, 50]. cfDNA derived from a tumor, also referred to as circulating tumor DNA (ctDNA), can be detected in a liquid biopsy such as blood, and allow for earlier detection, help classify a lesion, inform on mutational burden, and provide real-time disease monitoring in response to treatment [51–54]. We previously optimized a protocol to detect the dominant driver mutations in UM, especially wild type and mutant GNAQ and GNA11 (c626A > T and A > C) [55]. With this, our group had conducted in vivo studies of ctDNA in a rabbit model of UM and a clinical study in a UM patient cohort, finding ctDNA in blood and aqueous humor correlated with growth, malignancy, and enabled earlier detection of UM and premalignant nevus [55]. Here we applied these methods to detect GNAQ/11 cfDNA released by a panel of UM cells in the presence or absence of MF treatment. Consistent with the cytotoxicity of MF, the release of wild type and mutant cfDNA increased in a concentration-dependent manner. An increase in cancer cfDNA could signal widespread cytotoxicity and successful treatment or be indicative of adaptive mechanisms resulting in resistant populations [37]. Because UM cells were able to repopulate a culture upon removal of a 20 µM concentration of MF, then the amplification of cfDNA observed in these cultures may be consequence of actively secreted DNA [56]. Conversely, the large increase in cfDNA observed upon 40 μM MF treatment is most likely consequence of widespread cell death only, as UM cells were no longer capable of repopulating a culture plate upon drug removal. Importantly, the dose-dependent increase in ctDNA detection following MF suggests that such an assay could be used through a liquid biopsy as a non-invasive monitoring tool of MF treatment response in patients.

MF acts through PR modulation having inhibitory effects on proliferation and cell cycle progression in hormone responsive tumors [20, 27, 57]. The current reservoir of knowledge on PR expression in UM is scarce, dated, and contradictory [58, 59]. Questioning the relevance of PR to drive the observed effects of MF in UM, we sought to update the field and found that the panel of UM cells here studied does not express classical nuclear PR. However, as progesterone has functional affinity also for non-classical receptors, it is likely that MF may similarly have widespread functionality via such receptors [60–62]. Expanding our search we found that all non-classical surface progesterone receptors PAQR8, PGRMC1, PGRMC2, as well as the other known receptor for MF, the glucocorticoid receptor NR3C1, were present in the UM cells. Interestingly, PAQR8 and PGRMC1 where found stimulated by progesterone and associated with anti-apoptotic signaling cascades [63, 64]. PGRMC1 has been involved in a multitude of other cancer associated signaling pathways [64]. In vitro studies of uterine sarcoma and cervical cancers have demonstrated PGRMC1 to enhance the epithelial mesenchymal transition phenotypes, promote chemoresistance, and have a possible role in progression of metastasis [65, 66]. PGRMC2, similar to PGRMC1, have been implicated in different cancer signaling cascades, yet with likely tumor suppressor properties [60, 67, 68]. Of interest, in the UM cells studied here, 20 μM MF treatment resulted in the downregulation of PAQR8, PGRMC1, PGRMC2, and NR3C1. The later gene, which encodes for the glucocorticoid receptor, is of interest; most effects of the glucocorticoids are mediated by the alpha isoform of the glucocorticoid receptor (reviewed in [69]); however, we have shown that cells devoid of mRNA for the alpha GR isoform but expressing the beta mRNA isoform still respond to MF with growth inhibition [28]. Therefore, we cannot exclude that MF may elicit its anticancer effect targeting the beta isoform of the glucocorticoid receptor, which however seems to operate as a dominant negative regulator of the traditional alpha isoform [70, 71]. Finally, another exiting option behind the mechanism of action of MF to explore in UM is its capacity to induce stress of the endoplasmic reticulum while blocking the growth of cancer cells as we have shown in ovarian cancer [72]. Further studies are therefore required to investigate whether MF is indeed functioning through non-classical means and the mechanisms by which selective receptor modulation is occurring.

The repurposing or repositioning of MF into the clinic for treatment of cancer, in this case UM, could be very rapid; this is due to the fact that the safety profile in humans has been already tested as the drug is currently approved for two clinical conditions: (1) to ameliorate the hyperglycemia associated with Cushing’s syndrome; and (2) to terminate early pregnancies when accompanied with a prostaglandin analogue (reviewed in [27]). We anticipate that the concentrations of MF needed to be reached in vivo to inhibit functionality and growth of UM cells are achievable. According to pharmacological studies done in patients when MF was administered orally in doses up to 800 mg, the drug reached blood concentrations of up to 20 µM [73–76]. We provide evidence that concentrations higher than 20 µM not only impair functionally and arrest UM cells, but also kill them. However, in order to reach such concentrations in the circulatory system, either new derivatives of MF with higher potencies need to be synthesized, or new formulations of the drug, such as MF-carrying nanoparticles, should be developed in order to deliver higher amounts of MF into the microenvironment of the UM.

## Conclusion

This report is the first to investigate the anti-cancer effects of MF in the context of UM. Our results demonstrate potent growth inhibitory and lethal effects of MF on primary and metastatic UM cell lines in a concentration-dependent manner. These effects seem to be independent of cognate PR as no mRNA expression was detected for this receptor in any of the UM cell lines studied. The lethal effect of MF occurred in association with increased Annexin V-FITC/ PI double-labelled cells, DNA fragmentation, and caspase-3/7 activation, all consistent with the induction of apoptotic cell death. Of novelty, cfDNA levels of wild type and mutant copies of critical UM genes were recorded under MF treatment proving that a significant increase in DNA release occurs when MF is used at lethal concentrations. MF is a safe FDA approved drug with promising potential as a potent anti-cancer treatment. Repurposing MF would be a cost-effective means of finding new treatment options for patients with UM.

## Supplementary Information

**Additional file 1: Fig. S1** Depiction of confluency as assessed using the Incucyte software. Representative are masked images of MF41 cells treated with the indicated concentrations of MF for 72 h.

**Additional file 2: Fig. S2** Assessment of growth of wild type choroidal melanocytes (wtCM) (**A**) or mutant CM (mutCM) (**B**) in the presence or absence of 20 µM MF. Right panels in (**A**) and (**B**) represent the percent confluence of the cells in the absence of presence of MF. MF20: 20 µM MF; VEH: vehicle.

**Additional file 3: Fig. S3** Representative cell cycle histograms of UM cell lines exposed to vehicle or MF at 20 µM or 40 µM concentrations. Results were generated using the Guava Muse microcytometer. Colored in dark green are the hypodiploid DNA contents (a.k.a. Sub-G1 regions). Cells in G1 phase are colored in blue, those in S phase in red, whereas the light green represents the cells having G2/M content plus hyperploid DNA.

**Additional file 4: Fig. S4.** Caspase-3/7 activity in wild type CM (wtCM) (**A**) or mutant CM (mutCM) (**B**) exposed for 60 h to vehicle (VEH) or 40 µM MF (MF40). Left panels in (**A**) and (**B**) show phase contrast images, whereas the middle panels represent the staining denoting caspase-3/7 activity; the quantitation of the activity of caspase-3/7 is depicted in the right panels.

**Additional file 5: Fig. S5** Overlay images of phase contrast with green fluorescence representing nuclear regions within the cells that accumulate a product of the enzymatic activity of executer caspase-3 7. These images represent the same fields shown in Fig. 5.

## Data Availability

Not applicable.

## Abbreviations

ANOVA: Analysis of variance
BAP1: BRCA-1 associated protein 1
cfDNA: Cell free DNA
ctDNA: Circulating tumor DNA
cdk: Cyclin-dependent kinase
ddPCR: Droplet digital polymerase chain reaction
EIF1AX: Eukaryotic translation initiator factor 1A X-linked
GNAQ: Guanine nucleotide-binding protein G subunit alpha
GNA11: Guanine nucleotide-binding protein G subunit alpha 11
PGRMC1: Membrane-associated progesterone receptor component 1
PGRMC2: Membrane-associated progesterone receptor component 2
MF: Mifepristone
CM: Choroidal melanocytes
PI: Propidium iodide
PR: Progesterone receptor
PAQR8: Progestin and adipoQ receptor family member 8
qPCR: Real time polymerase chain reaction
NR3C1: Receptor subfamily 3 group C member 1
UM: Uveal melanoma
