# Supplementary figures and images for: Anticancer effects of mifepristone on human uveal melanoma cells
Source: Cancer Cell Int. 2021 Nov 17;21:607. doi: 10.1186/s12935-021-02306-y (PMC8597220; doi:10.1186/s12935-021-02306-y)

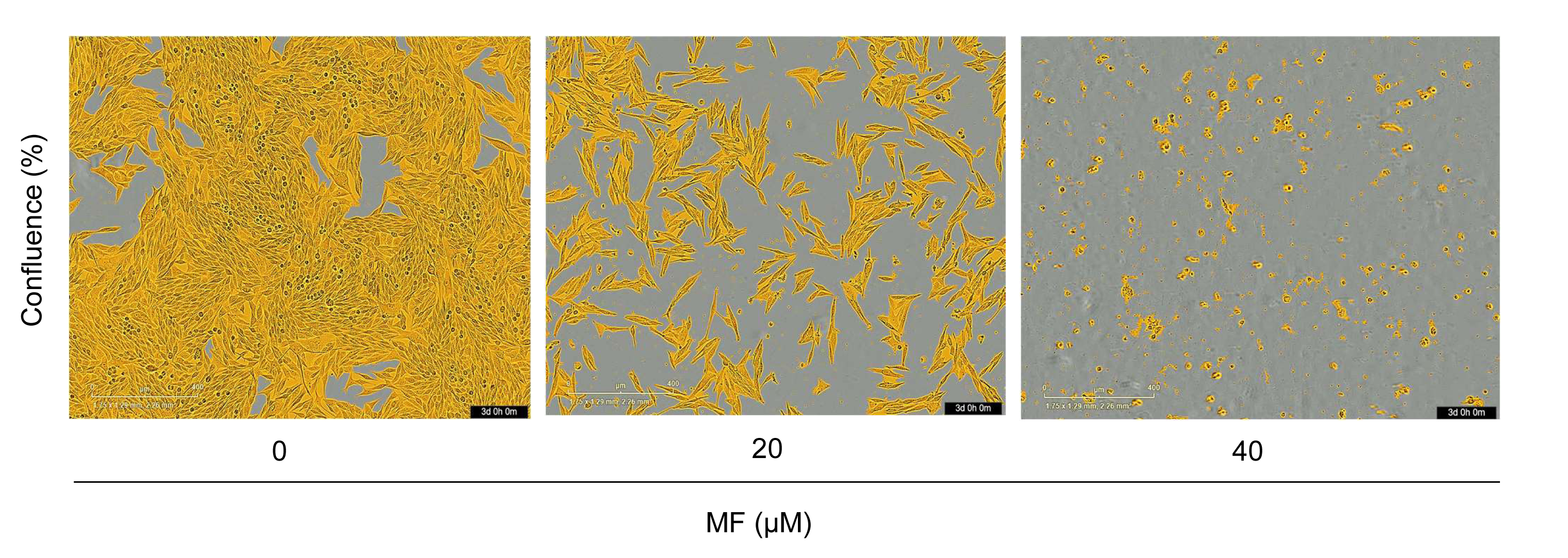

Supplement: Supplementary file 1 — Additional file 1: Fig. S1 Depiction of confluency as assessed using the Incucyte software. Representative are masked images of MF41 cells treated with the indicated concentrations of MF for 72 h. [file 12935_2021_2306_MOESM1_ESM.tif]

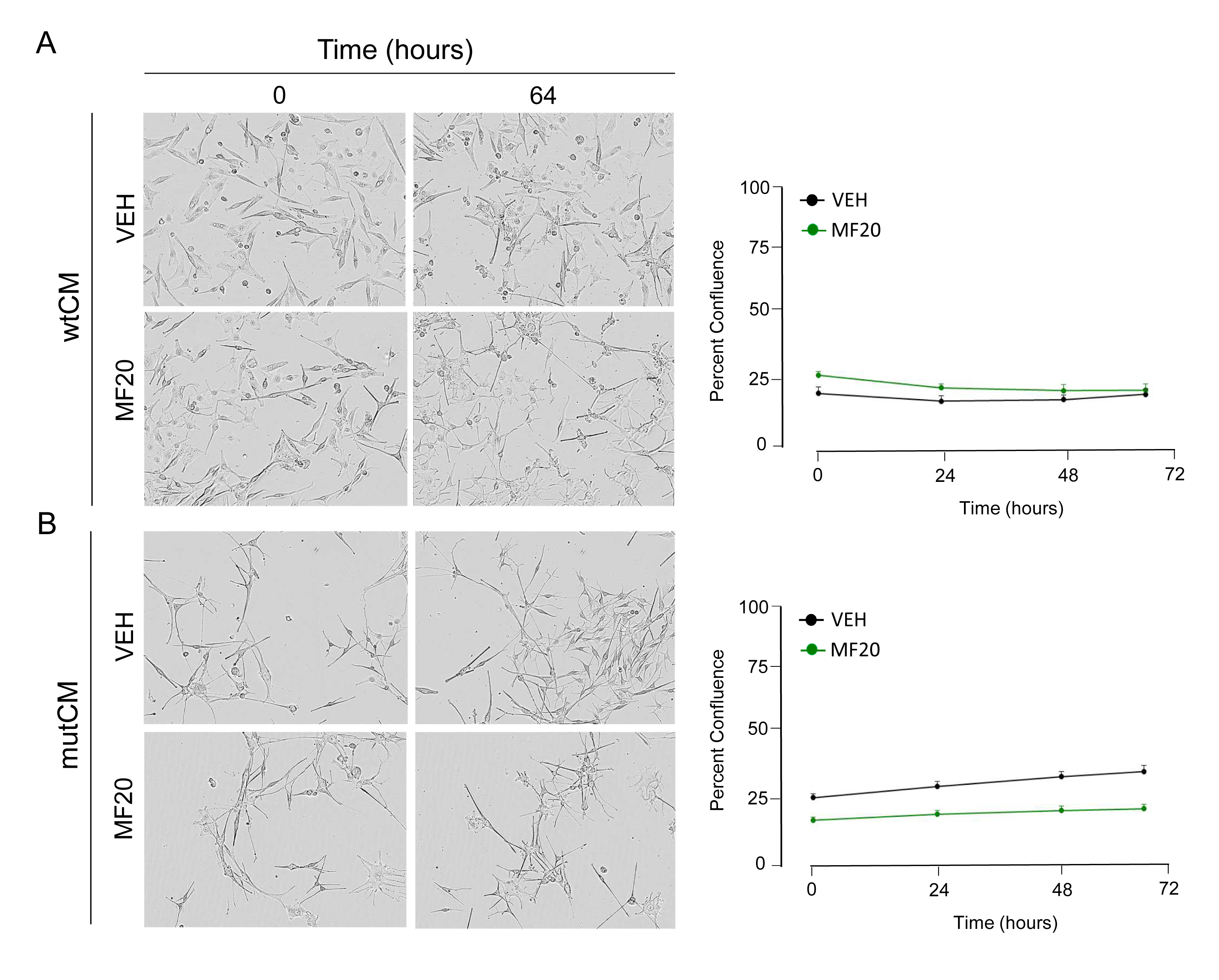

Supplement: Supplementary file 2 — Additional file 2: Fig. S2 Assessment of growth of wild type choroidal melanocytes (wtCM) (A) or mutant CM (mutCM) (B) in the presence or absence of 20 µM MF. Right panels in (A) and (B) represent the percent confluence of the cells in the absence of presence of MF. MF20: 20 µM MF; VEH: vehicle. [file 12935_2021_2306_MOESM2_ESM.tif]

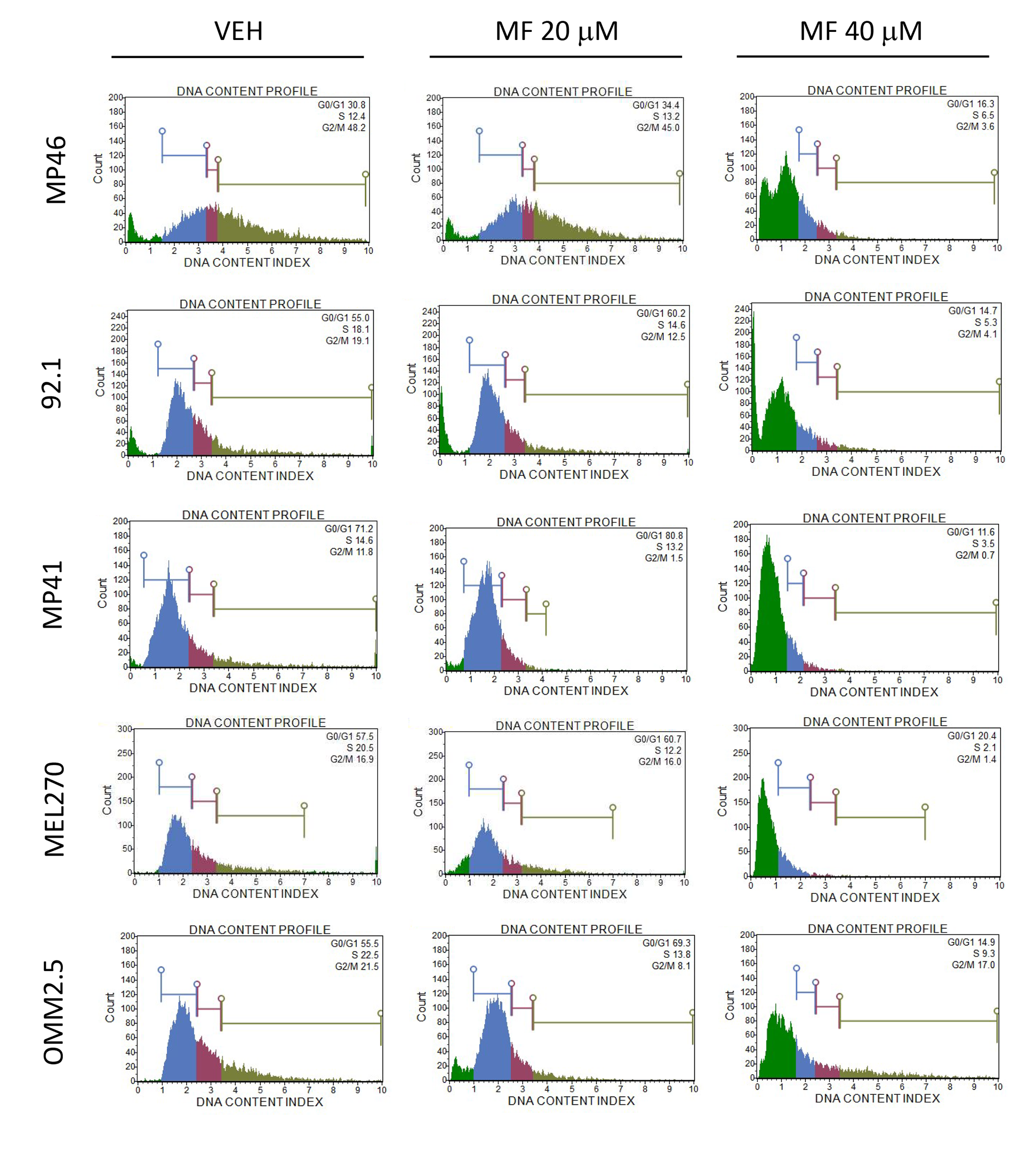

Supplement: Supplementary file 3 — Additional file 3: Fig. S3 Representative cell cycle histograms of UM cell lines exposed to vehicle or MF at 20 µM or 40 µM concentrations. Results were generated using the Guava Muse microcytometer. Colored in dark green are the hypodiploid DNA contents (a.k.a. Sub-G1 regions). Cells in G1 phase are colored in blue, those in S phase in red, whereas the light green represents the cells having G2/M content plus hyperploid DNA. [file 12935_2021_2306_MOESM3_ESM.tif]

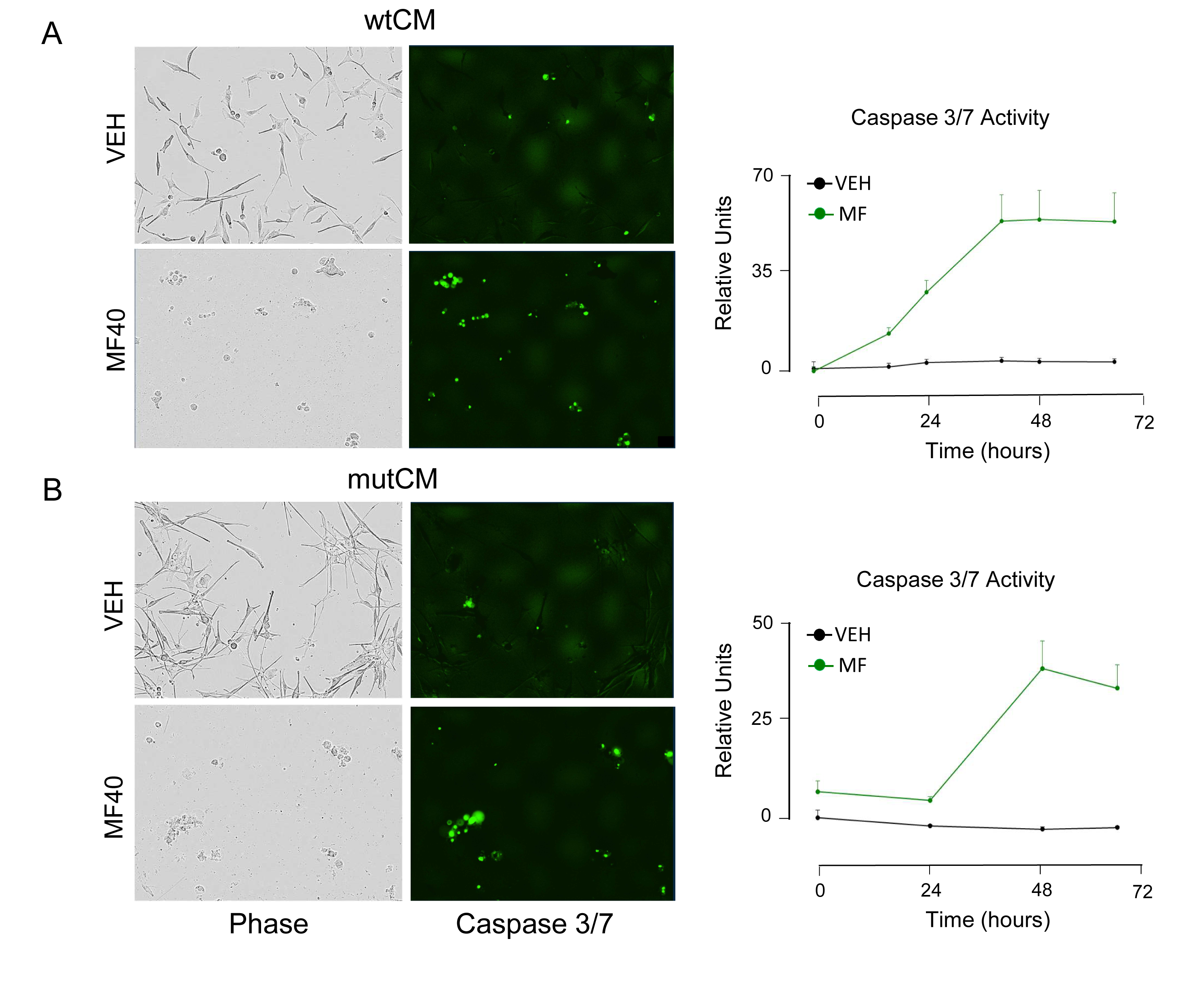

Supplement: Supplementary file 4 — Additional file 4: Fig. S4. Caspase-3/7 activity in wild type CM (wtCM) (A) or mutant CM (mutCM) (B) exposed for 60 h to vehicle (VEH) or 40 µM MF (MF40). Left panels in (A) and (B) show phase contrast images, whereas the middle panels represent the staining denoting caspase-3/7 activity; the quantitation of the activity of caspase-3/7 is depicted in the right panels. [file 12935_2021_2306_MOESM4_ESM.tif]

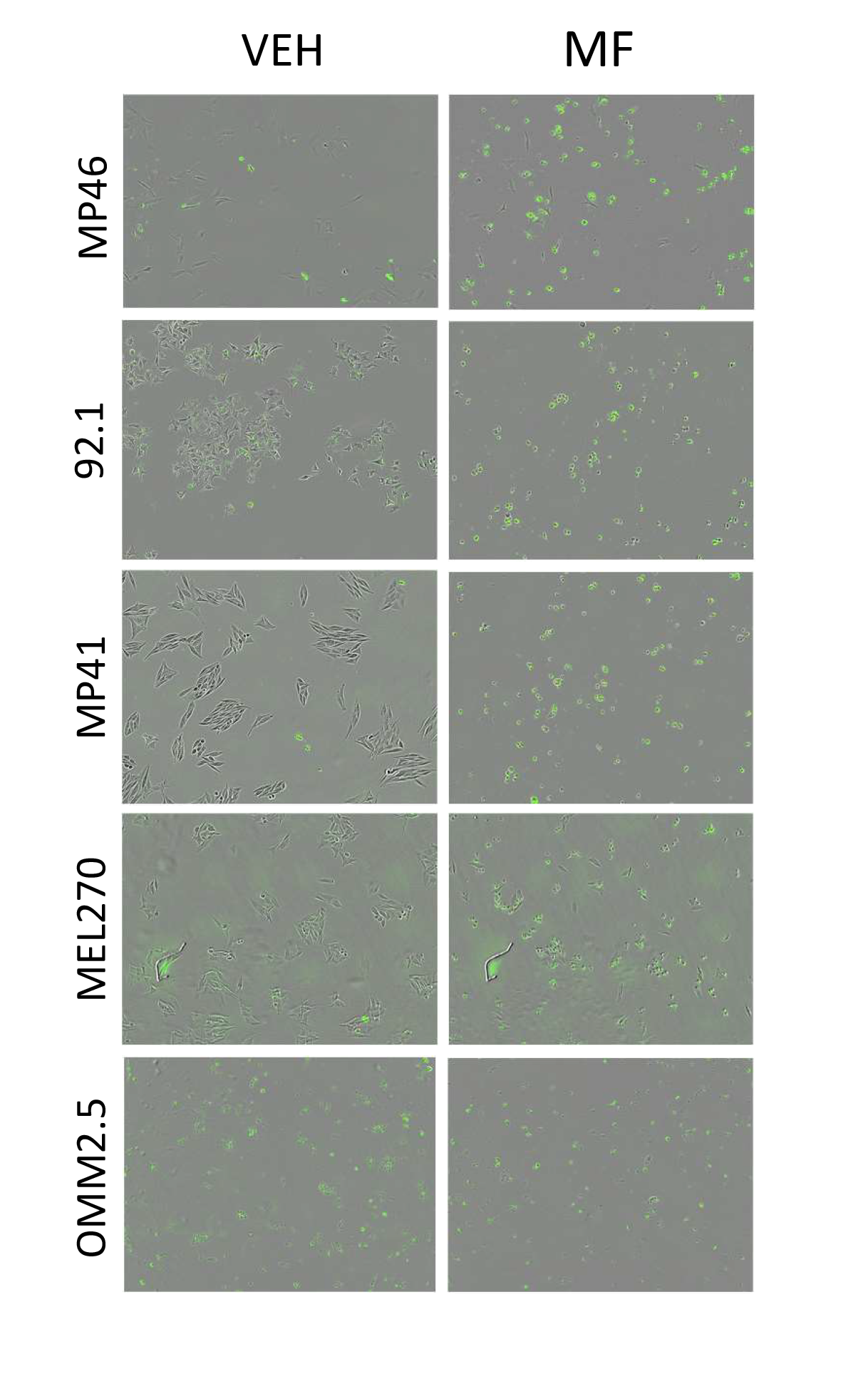

Supplement: Supplementary file 5 — Additional file 5: Fig. S5 Overlay images of phase contrast with green fluorescence representing nuclear regions within the cells that accumulate a product of the enzymatic activity of executer caspase-3 7. These images represent the same fields shown in Fig. 5. [file 12935_2021_2306_MOESM5_ESM.tif]
